# Developing Requirements for a Standardized System to Return Individual Research Results Back to Study Participants: Narrative Review

**DOI:** 10.2196/65606

**Published:** 2025-08-18

**Authors:** Rosalyn Leigh Carr, Vita Chan, Nicholas C West, Matthias Görges

**Affiliations:** 1 The School of Biomedical Engineering, University of British Columbia, 6088 University Boulevard, Vancouver, BC, V6T 1Z3, Canada, 1 6048227810; 2 Research Institute, BC Children's Hospital, Vancouver, BC, Canada; 3 School of Information, University of British Columbia, Vancouver, BC, Canada; 4 Department of Anesthesiology, Pharmacology and Therapeutics, University of British Columbia, Vancouver, BC, Canada

**Keywords:** return of results, research results, knowledge translation, system, researching, researcher, research participants, study participants, personalization, data integration, data visualization, research ethics, narrative review

## Abstract

**Background:**

The increasing prevalence of smart devices has created vast amounts of untapped data, presenting new opportunities for data sharing across various fields, such as environmental sciences, health management, and astrophysics. While a significant portion of the public is willing to donate personal data, we need to better understand how to obtain information about which data assets a person may hold and the risks, benefits, and potential uses of this data exchange mechanism. Developing a trusted data-sharing platform may increase participants’ willingness to donate data and researchers’ ability to return personalized results from research findings.

**Objective:**

This study aimed to develop a preliminary list of core requirements, which can be used to develop design recommendations for standardizing the return of individual research results to study participants across research disciplines.

**Methods:**

We conducted a narrative literature review of existing platforms used to return research results to study participants. The search strategy included English-language articles published between May 2013 and May 2023. Concepts related to returning, disseminating, and sharing research results were searched for in (1) published research reports on Web of Science and MEDLINE, (2) gray literature, and (3) the bibliographies of included articles. Screening and data extraction were performed by 2 independent reviewers using Covidence. Inclusion criteria required that the study (1) included human participants, (2) returned information based on data collected from or by participants, (3) was published in English, and (4) included a description of a results-sharing system. Articles that met all 4 inclusion criteria were included in the review; articles that met the first 3 were also presented as supplementary articles. Results and requirements were synthesized thematically.

**Results:**

Overall, 6608 abstracts were screened, and 266 articles underwent full-text review to identify 8 articles describing the development and evaluation of 7 different return of results systems. In total, 7 of the 8 articles reported the use of multimodal dissemination methods, including a combination of physical documents, emails, phone calls, and digital platforms to support text and graphical data representations. One article outlined accessibility features to serve the specific participant population. None of the articles described in detail how results were or were not anonymized. A total of 4 studies relied on an expert or clinician to share results on behalf of the research team. Additional educational or contextual materials were included alongside results in four studies, including specific materials designed for follow-up with experts and clinicians. Participants were not hesitant to receive unfavorable results and instead aimed to incorporate such information into their lives via lifestyle changes, clinical intervention, or seeking community.

**Conclusions:**

Return of results systems should support multiple modes of dissemination for text-based results. Additional educational and lay-language materials are helpful for participants to understand and use information gained from receiving results.

## Introduction

### Background

In 2019, it was estimated that almost 70% of households had a smart device or appliance [1], and as of 2021, 85% of North Americans owned and used a smartphone [2]. This proliferation of smart technology has generated vast amounts of largely untapped data, presenting tremendous new opportunities for open science and knowledge sharing—particularly in areas where citizen scientists [3] want to contribute to academic discovery and applied research. Examples include water quality [4], astrophysics [5], environmental sciences [6], and patient-generated health and activity data [7], which can affect patients’ health management by enhancing their ability to convey their health status [8] and improving patient-clinician relationships [9].

As with biobanks, where people can donate samples of tissues or body fluids for targeted or unrestricted use in research and health care [10], there is growing interest in donating personal data in various research areas [11]. Examples include a Swedish activity and physiology data sharing app [12], the ecobee smart thermostat “donate your data” dataset [13], or Tidepool’s continuous glucose monitor “Big Data Donation Project” [14]. Increasing public trust may expand the use of this approach.

There is growing support for citizens to be able to donate their data for research. In a United Kingdom survey of 1300 members of the public, 54% of respondents were willing to donate personal data for research [15], motivated by both self-benefit and altruism [15]. However, barriers to data donation include a need to better understand the risks and benefits associated with their data and its potential uses, as well as gain insights into which data assets a person may hold [16,17]. Combined with privacy concerns regarding data-sharing methods, this highlights the need to create trusted data-sharing platforms. Similarly, there may be an enhanced willingness to share data if there is perceived self-benefit, such as the ability to derive insights from one’s data or benchmark results against others, which could help their interpretation and provide locally relevant context [18].

Acknowledging these challenges, the National Academies of Sciences, Engineering, and Medicine assembled a committee to develop comprehensive guidance on returning individualized research results from biospecimens [19]. This report outlines 12 key recommendations to support study-specific decision-making, enhance research quality, improve participant understanding, and align with current regulations.

The recommendations emphasize the significance of considering the value of results to participants, evaluating the quality of research laboratories, and engaging community and participant representatives in policy development [19]. They also advocate providing adequate resources for high-quality research, planning for result return in funding applications, and supporting research to broaden the empirical evidence base. In addition, the recommendations call for revising regulations to facilitate the return of individual research results, harmonizing definitions across federal regulations, and ensuring transparency during the consent process. Finally, while the report addresses various factors influencing the feasibility of returning individual results to participants, including the potential burdens that investigators face when doing this for a single study, it does not discuss the systems available for doing so at scale (ie, by establishing infrastructure to be leveraged across multiple studies) or their potential impact on the widespread adoption of practices to return individualized results to study participants.

Existing studies on the return of results have likewise focused on policy, guidance, and best practices [19-22], or the perceptions of both participants and researchers regarding the return of results [23-26]. However, there has not been a formal review of the tools and methods used to return results, as many studies are restricted solely to their relevant discipline and data. This study aims to expand on this discussion.

Some fields have existing context-specific platforms to return research results. Environmental sciences have the digital exposure report-back interface, which returns chemical exposure results, such as lead contamination in drinking water [27], and Gardenroots, which illustrates contaminant levels in water, soil, and plant samples contributed by participants [28]. There are recommendations for returning environmental health-related data results [29], but other fields have less well-defined mechanisms [30].

### Objective

This study aimed to develop a preliminary list of core requirements, which can be used to develop design recommendations for the standardizing of the return of individual research results to study participants across research disciplines.

## Methods

### Study Design

We conducted a narrative review of the published literature for reports of existing platforms used to return research results to study participants. The protocol was registered at the Center for Open Sciences’ registry on May 24, 2023 [31]. A narrative review was chosen because the breadth and heterogeneity of this topic made a systematic review impractical. Systematic reviews are ideal for narrowly focused questions and structured data synthesis. In contrast, narrative reviews allow for broader interpretation and integration of diverse sources, which was essential for our research aims [32]. We followed the registered protocol for article selection but extended it to present a larger set of articles that met a limited set of our inclusion criteria as supplementary material.

### Literature Search Strategy

Terms related to research results, result dissemination, and sharing of individual data from studies were used to search the literature. The search method combined search terms using proximity searching to identify key phrases related to the methods and process of returning research results. This approach was chosen instead of traditional keyword searching as the terms describing the method of returning research results to participants were often not specific enough to avoid large quantities of extraneous articles. These included cases of “research” being used as an adjective alongside “data” or “results,” while similarly, the term “results” tended to include headers and titles within abstracts rather than methods. An initial search strategy was created with the guidance of a research librarian to identify key phrases to search in abstracts, titles, and keywords (Multimedia Appendix 1).

A search was performed on Web of Science (Appendix A) and MEDLINE (Ovid; Appendix B). All searches were limited to articles published between May 2013 and May 2023. Gray literature was also searched by including the first 100 results with the search term “returning research results to participants” on Google Scholar. IEEE Xplore was also searched using this phrase and others, as multiple proximity operators are not supported within the same search. Finally, the reference lists of all identified reports and articles were iteratively searched for additional studies until no more relevant articles were identified.

All articles were uploaded into Covidence (Veritas Health Innovation, Melbourne, Australia), and duplicates were removed.

### Article Selection Process

Two reviewers (RC and VC) independently completed an initial screening based on the article titles and abstracts to identify which articles would be included in the full-text review. The inclusion criteria were: (1) the reported study included human participants (members of the public or nonexperts) receiving information on their study results; (2) the information returned to participants was based on data previously collected from or by participants and included at least one variable with a description of the variable (ie, what was being measured) and domain context (ie, the meaning of this measurement in the context in which the study was conducted); (3) the article was published in English; (4) the article must include a description of a system or design (implemented as a pilot, at least) for sharing research results. Articles that met all 4 inclusion criteria were included in the full review, while articles that met the first 3 inclusion criteria but not the fourth were included as supplementary articles.

Two reviewers (RC and VC) then independently performed a full-text screening of the selected articles. Disagreements were resolved through discussion; if there was no resolution, additional reviewers were added to achieve consensus. Articles meeting criteria (1), (2), and (3), but not (4), formed a larger pool of supplementary articles of secondary interest. Articles that met all 4 inclusion criteria, including the additional system criteria, were selected to undergo full data extraction.

### Data Extraction

Two independent reviewers (RC and VC) used a standardized data extraction form in Covidence to extract data from each selected article (Multimedia Appendix 2). The following information was extracted into the template: study details, study characteristics, population characteristics, data sharing methodology, and data characteristics.

### Risk of Bias and Quality Assessment

Two researchers (RC and VC) independently rated the quality of each study using the Authority, Accuracy, Coverage, Objectivity, Date, Significance checklist [33], as none of the included articles included randomized control trials, which was a prerequisite for using other tools.

### Data Analysis

Data collected were analyzed and synthesized by 2 researchers (RC and VC) using thematic analysis [34]. Open coding was initially used to identify key segments in the data, after which themes were developed inductively through an iterative process. These themes were then organized into a hierarchy to form key findings. The findings include summarized information on study characteristics, variables measured, data presentation, delivery mechanism, results contextualization, whether the data shared are personalized, and system design requirements.

## Results

### Study Selection

**Figure 1.**
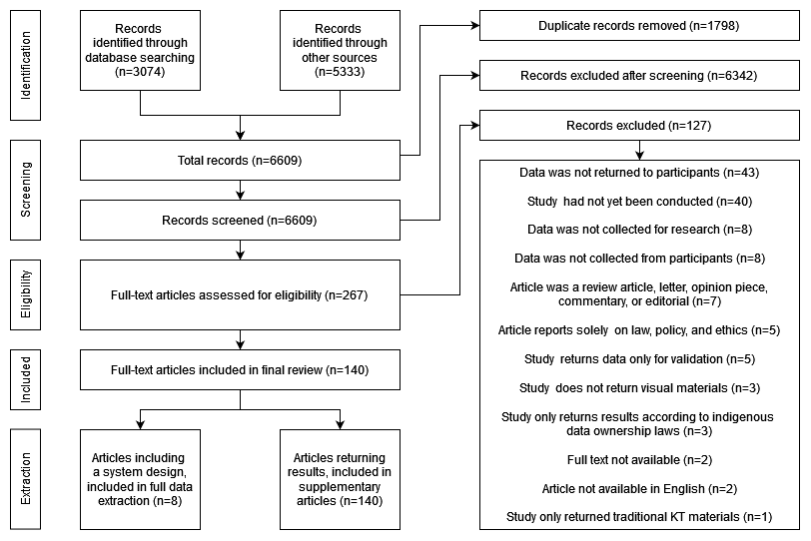
Study selection and inclusion flow chart using Preferred Reporting Items for Systematic Reviews and Meta-Analyses (PRISMA) categories. All articles included in the full data extraction are also included in the supplementary articles, as they meet the inclusion criteria for both groups.

After removing duplicates, 6609 articles were identified from the literature search and bibliographies of iteratively included papers. After reviewing the titles and abstracts, 267 articles were selected for full-text review. The total number of articles included in the supplementary list was 140 (Multimedia Appendix 3). From this, 8 articles [27,35-41] met all 4 inclusion criteria; however, 2 articles described a single study, discussing its design criteria in 1 article [27] and its implementation in another article [40], which were combined, resulting in a total of 7 studies (Figure 1).

### Study Characteristics

The eight included articles (Table 1) were published between 2017 and 2023, with 5 articles from the health care domain [35-39] and 3 (describing 2 studies) from the environmental health domain [27,40,41]. In total, 4 studies were based in the US [27,35,38,40,41], 1 in the Netherlands [39], 1 in the UK [37], and 1 in Japan [36]. The total number of study participants varied from 20 up to 24,163, with 1 study including 7 researchers as additional participants to provide feedback on system usability [41].

In total, 5 of the 7 studies involved vulnerable groups, including children [27,40,41], infants and mothers [38], retinal disease patients with significant vision loss [37], and cancer survivors [39]. In 2 studies, participants were identified and recruited from previously collected data (previous genetic testing or whole genome sequencing) [35,36]. In total, 4 studies included recruitment directly through a health care provider (HCP) or hospital setting [35,37-39], with 1 of these studies being a company recruiting via local HCPs [37]. In 1 study, the primary participants were researchers [41], and in 1 study, participants were recruited through community engagement or door-to-door soliciting [27,40]. Financial compensation was provided for participation in some study activities in 1 study [38].

**Table 1.** Study characteristics of the seven studies including system design.

Study	Study domain	Participant characteristics	Sample size	Country or region
Boronow 2017 [27]; Perovich, 2018 [40]	Environmental health	Children with doctor-diagnosed asthma	10 participants completed feedback interview	US; Metro-Boston (Lowell, Lawrence, Old Colony, Castle Square) and Cincinnati
Cope, 2023 [38]	Health care	Babies of birthing parents at least 11 years of age	1823 participants completed feedback survey, 24 participants completed feedback interview	North Carolina
Gilbert, 2022 [37]	Health care	Patients with inherited retina disease	20 participants attended initial focus groups, 30 participants attended technology workshops, 50 participants attended user research focus groups	UK; London
Ohneda, 2022 [36]	Health care	Population-based genome cohort	161 participants completed questionnaire survey before receiving results, 150 participants completed questionnaire survey after receiving results	Japan
Polka, 2021 [41]	Environmental health	Homes in two environmental justice communities, children in areas with manganese in their local drinking water	7 researchers interviewed	US; Massachusetts
van de Poll-Franse, 2022 [39]	Health care	Cancer patients or survivors	N/A^a^ (population registry)	Netherlands
Savatt, 2018 [35**]**	Health care	Anyone who has had genetic testing regardless of genetic test results or diagnosis	1601 participants enrolled in GenomeConnect	US

a not applicable.

### Risk of Bias and Quality Assessment

Based on the Authority, Accuracy, Coverage, Objectivity, Date, Significance checklist (Multimedia Appendix 4), none of the seven studies had a strong indication of bias, with 3 having minor concerns.

### Method of Dissemination

Several modalities of returning results were reported, with seven articles describing the use of 2 or more methods. Results were returned as a physical report through traditional mail in 2 studies [36,40], and 1 also returned results at an in-person community meeting [40]. A total of 4 studies used an online web-based portal [35,36,38,39], and 1 used a dedicated mobile application with specific accessibility features for people with vision loss [37]. Finally, 1 study used an online portal that required a clinician to deliver specific results (such as positive screening results) by phone [36] or an unspecified mechanism [38]. In total, 2 studies required clinicians to review before dissemination, though the clinicians did not disseminate results directly [35,37]. Emails were used by 2 studies to notify participants that their results were available [35,38] or that changes had been made (such as a new interpretation of previously collected data) [35].

### Data Format

Out of 7 studies, 6 studies returned at least part of the participants’ results in a text format (written word) [27,35-38,40,41] (Table 2), with 1 article not specifying the data format used [39]. Visuals were also included in 4 studies as charts or graphs [27,40,41], image data (such as retinal scans) [37], or graphics (non-data images used for aesthetic purposes) [36]. A total of 3 studies created their own results report for their participants [27,36,40,41], 1 returned standard medical laboratory reports [38], and 1 created a specific “physician report” alongside the participant report for sharing with their HCP [36].

In addition, 1 study also included nontraditional results in the form of personalized t-shirts for participants that included the number of chemical values measured in their homes and aggregate results in the form of data physicalization [42], which participants could observe at a community meeting organized by researchers [40].

**Table 2.** Data characteristics of the seven studies including system design.

Study	Presentation of information	Mechanism of delivery	Contextualization of results	Identifiable	Education of results
Boronow 2018 [27]; Perovich, 2017 [40]	Two report-back packets designed with the DERBI system containing personalized graphs and text summaries, personalized data shirt, and data physicalization (physical objects that display data in the real world instead of on paper or a screen)	The two report-back packets were delivered in person during a home visit and a community meeting. Personalized data shirts and data physicalization were available to participants who could attend the community meetings. Participants who could not attend were mailed their report-back packets.	In addition to personalized results, participants received aggregate results of chemical levels in the homes of other participants in the study cohort.	Personalized	Yes
Cope, 2023 [38]	Portal results page explains the meaning of a normal result, accuracy of the screening test, and information about the diseases that were screened. CLIA-certified laboratory report provided.	Participants with normal results were notified via email when results were available on the portal. Participants with positive screening results were notified by the study genetic counselor.	No context	Personalized	Yes
Gilbert, 2022 [37]	Not reported	MyEyeSite patient application	Not reported	Personalized	
Ohneda, 2022 [36]	Participants received information about generic and brand names of drugs considered as potential risks, along with their indication, medicinal effects, and individual predicted genotyped-based response to the drug. Health professionals of patients were given a single sheet containing all three genes, medication, and dosage recommendations based on the genomic results	Mail and genetic counseling through telephone	No context	Personalized	Yes
Polka, 2021 [41]	Numerical, text, and graphical representations of participant’s results	Not reported	Population and study cohort	Personalized	Yes
van de Poll-Franse, 2022 [39]	Not reported	Not reported	Population; age- and sex-matched normative population with cancer	Personalized	Yes
Savatt, 2018 [35]	Notification with general statement that genetic test results have been updated and suggestion to contact the ordering healthcare provider or genetics provider in their area	Email	Match with other participants with similar characteristics (eg, gene, disease, U.S. state)	Not reported	Not reported

### Contextualization, Education, Recommendations, and Results Literacy

In 4 studies, additional details were provided alongside returned results to contextualize the information and provide recommendations [27,36,38,40,41]. One article (which described 2 pilot studies) described the return of individual in-home air concentrations of common contaminants, accompanied by general information on common pollutant sources and population averages for comparison [41]; the second pilot study returned individual tap water manganese levels, including general information and “group-level information,” likely aggregated data from the study population, but which was not specified [41]. Another study included additional information on diseases for which participants had been screened in the results [38]. Other studies included recommendations about “improving” one’s results (lowering exposure to specific chemicals) [40] or recommendations for avoiding specific drugs based on one’s pharmacogenetic results within the results [36].

### Expert Intervention, Follow-Up, and Community Engagement

Experts, primarily clinicians such as genetic counselors, were involved in 4 studies at various stages [35-38]. In 2 studies, clinicians reviewed results before dissemination [35,37], and 2 required a clinician to deliver specific results [36,38]. Genetic counseling was mandated for participants with specific results in one study [36]. Finally, 1 study, which allowed participants to upload data directly to the application, would automatically notify an HCP if data exceeded predefined thresholds so that an intervention could occur [37].

Follow-up with experts outside of the study team was also common: 2 studies nudged their participants to follow up with their primary HCP [35,36], one of which had prepared and included tailored results for this purpose [36]. Participants also expressed their plans to share their results with their HCPs [37,38,40]. However, this motivation was sometimes mediated by the results, as participants in 1 study did not feel the need to report “normal” results to experts [38].

When participants were not directly engaging with experts, one platform also provided methods to engage with additional research opportunities and clinical trials [37]. In contrast, another study looked at creating or maintaining a community involved in the research through community events and engagement sessions [40]. One platform also allowed participants to view relevant products to purchase [37].

### Identifiability and Anonymity

The extent to which data or results were identifiable was not clearly stated in all 8 articles. Researchers in 1 study used participant IDs in the internal database that were only accessible by researchers to avoid linking identifiers [36]. Another system only made information accessible to researchers that could not be easily identifiable (such as unlabeled images or appointment schedules) [37]. The methods were unspecified in one article, though data were described as “anonymous” [35] and 2 articles did not specify any level of inherent anonymization unless researchers deliberately did this using outside tools or methods [27,41]. Finally, 1 article did not mention privacy or anonymity, but the registry was tied to the local government and health authorities [39].

### Participant Perceptions

Participant perceptions of results were positive, with sentiments of increased trust, autonomy, and knowledge being key factors [27,37,39,40] (Table 3). In 1 study, participants did not experience worry or anxiety associated with receiving results [27,40]. Negative perceptions included a lack of adequate education materials or appropriate language [36,38], inadequate anonymization and options to opt-out of communications [37], or not having the option to receive additional results if they became available [35].

**Table 3.** Feedback and conclusions of the seven studies including system design.

Study	Positive participant opinions	Negative participant opinions	Researcher opinions
Boronow 2018 [27]; Perovich, 2017 [40]	Motivated to join because participants are able to access information that wouldn’t be available otherwise. Participants felt like they were able to build trust with researchers. Results did not cause excess worry/anxiety/distress to participants. Participants made specific positive changes and brought study results to medical resources.	Participants recommend creating report-back materials for different audiences.	Digital exposure report-back interface is good for large studies where personalization is very important.
Cope, 2023 [38]	Calling participants with positive screening results ensures that participants with significant implications for their health are aware. The portal was rated easy to use and helpful for understanding individual research results. But literature indicates that online portals may be only acceptable to some participants.	Avoid technical terms and use plain language as much as possible.	Returning individual research results online was a time-efficient method that reduced burden on study staff.
Gilbert, 2022 [37]	Participants reported increased agency and being empowered to manage their own conditions. Information should be expressed in lay terms.	Patients who lack experience with digital tools rely on others for assistance. Data needs to be anonymized, and there should be easy opt-in/opt-out options for research studies and receiving notifications for eligible studies and findings from studies	—^a^
Ohneda, 2022 [36]	Participants’ knowledge of PGx testing improved after receiving results	Even though the basic information of PGx had been given several times, some participants felt that the PGx concepts and terminology were difficult to fully understand	—
Polka, 2021 [41]	—	—	Manual report-back allows researchers to become familiar with participants’ results, but the process is time-intensive, which limits individualization and creativity and presents challenges of data accuracy
van de Poll-Franse, 2022 [39]	Return of individual results not only empowers patients but can also be an incentive for cohort retention. Comparison to an age- and sex-matched normative population is helpful in the interpretation of findings	—	—
Savatt, 2018 [35]	—	Registry participants want to receive information about potential updates to their genetic testing results (ie, if the interpretation has changed)	—

a not applicable.

### Researcher Perceptions

There was 1 study that included interviews with the researchers who were tasked with creating and disseminating the results. Even with a dedicated system, researchers report that the process was time-consuming, particularly for individual results [41]. Another study that included interviews with the research team as well as the participants who received the results reported that researchers were concerned about the generalizability of the participants’ results and that participants would have poor reactions to receiving “bad” results [40].

### Supplementary Articles

There were 140 supplementary articles (Multimedia Appendix 3), including the 8 articles identified in the primary search. The inclusion of these articles illustrates the return of results more broadly beyond digital systems.

In total, 98 articles reported genetics studies, with 27 relating specifically to *BRCA*1 and *BRCA2*. A total of 20 articles came from environmental health, mostly related to chemical exposure, and 16 articles came from the general health domain. One study was related to staff retention, and another to family studies.

The return of aggregate results was described in 36 articles, the return of personalized results in 114 articles, and the return of nonidentifiable results in 3 articles. Additional education materials were included alongside results in 104 studies, while 11 articles did not report on this.

**Figure 2.**
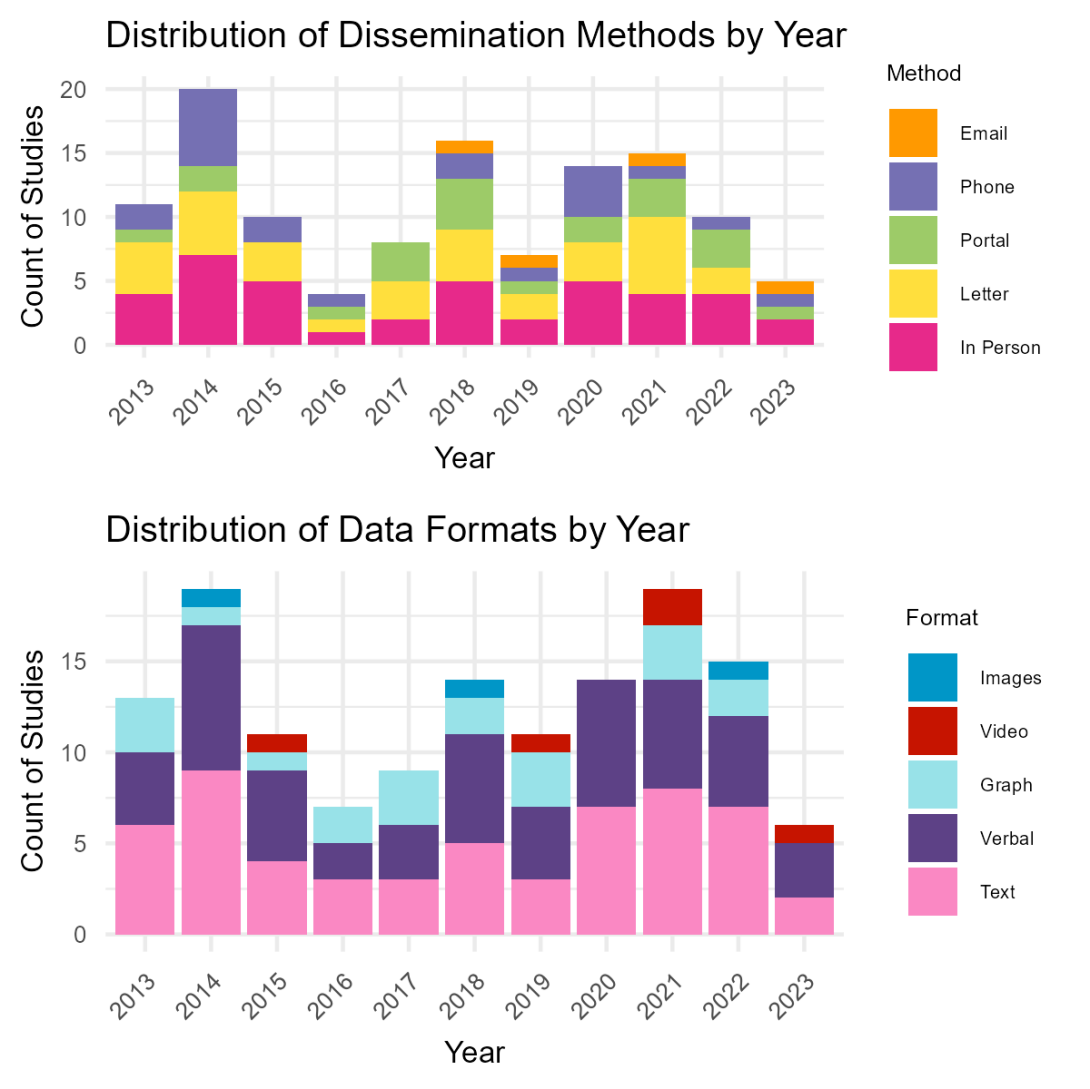
(Top) Distribution of dissemination methods of returned results (if reported) per publication year. (Bottom) Distribution of data formats of returned results (if reported) per publication year.

Of the possible methods for disseminating results either fully or partially (in cases where multiple methods were used), 56 studies delivered results via an in-person interaction, 48 used letters through traditional mail, 35 used phone calls, 25 used an online portal or application, 5 used emails for disseminating their results, 4 used other non-traditional methods, and in 32 articles the method used was unspecified. Digital methods, such as email and online portals, have become more common in recent years (Figure 2).

The data format also varied with many studies using multiple modalities: 85 studies returned results verbally, 73 in text format, and eight in video format (including YouTube videos or “relaxation tapes”). Some additional extraneous formats included t-shirts, folders, infographics, booklets, colour-in posters and comics, and various presentations, including conferences with an unspecified audience. More diverse methods have become more common in recent years (Figure 2).

In total, 29 studies included charts or graphs in their returned results (6 included graphics, images, or other visuals, including “visual aids,” and 26 did not specify the format used).

A total of 85 studies involved experts to return results: 79 studies involved clinicians, with 62 including genetic counselors. Experts used to return results in other studies included a medical geneticist, an oncologist, physicians, nurses, and other HCPs. Other nonclinician experts included health educators, licensed social workers, laboratories, community health workers, community leaders, public health authorities, and decision-makers.

## Discussion

### Principal Findings

This review identified 8 articles that described and evaluated 7 systems for returning results to study participants and a total of 140 studies in which results were returned to participants, with or without using a specifically designed system. Among the 8 articles, 4 studies had dedicated digital platforms [35,37-39] to create results, share results, or both, while 139 of the total 140 studies used less scalable and costly methods, such as in-person meetings and phone calls, to return some or all results to participants.

Many researchers and research ethics boards are concerned about providing adequate participant privacy [27], and this concern is particularly relevant when participants receive personalized results. Typically, including personal information is required for the information to be valuable to the participant (such as receiving personal genetic results instead of study-level results), yet some researchers opted to preserve participant privacy at the cost of the relevancy of the results [43]. There may be a mismatch between the privacy risk participants perceive versus the privacy risk researchers believe participants perceive, as researchers tend to self-censor when considering research methods, including the return of results [37,44-46]. For example, while many researchers would likely consider t-shirts with individual data for participants [40] to be a blatant misuse of private data and put participants at risk, such personalized tokens were well received by the participants in that study as it aided in them forming a community, not only with the researchers but amongst themselves [40]. Such non-traditional options may be permissible by ethical research frameworks but might not be considered due to researcher uncertainty around what is permitted and misconceptions of what may be beneficial to participants (such as the opportunity for peer support). While specific privacy and security measures are crucial, such as password-protected portals [38] or anonymizing data where possible [36], community benefits may be another factor to consider.

Dissemination modes constrain potential data formats. For example, returning graphical data over a phone call is impossible. The COVID-19 pandemic required a switch in modalities for all research fields [39,47-49], and more recent studies often required genetic counseling to be conducted over the phone rather than in person, where physical documents could be exchanged [36].

The accessibility of dissemination modalities should also be a consideration. Returning results in physical formats (such as physical documents, phone calls, or in-person meetings) often limits the ability to return results at scale due to resource limitations. However, transitioning to a fully digital system is not equitable for all participants for reasons including age, race, socioeconomic status, location, and language proficiency [50,51]. The ability to provide results in different formats facilitates the opportunity for participants to make use of the information for themselves [27,36,40]. This accessibility becomes even more crucial with participants from vulnerable populations, as they often have unique accessibility concerns [37], but also have increased incentives for getting information from research projects [37,38,40].

An additional consideration for creating accessible results is including adequate language and educational or contextual materials alongside results. While some participants may be well-versed in the research context due to previous research experience, most are laypeople. Therefore, considerations must be made regarding literacy and education [52]. Even when attempts were made to contextualize or explain complex concepts by providing additional educational materials, many participants still struggled to understand their results and what to do with the information [36-38]. While improving lay formats will always be valuable [53], additional prestudy or preresults educational materials may help bridge the gap.

### Key Requirements of a Results-Sharing System

First, it is crucial to support multiple dissemination methods, such as supplying physical documents alongside digital text [27,36,38,40]. Digital systems, such as secure portals, may require considerable development effort but offer easy-to-use and scalable solutions [35,38,41]. However, additional physical formats like mailed documents should be available to participants as an option for the few who require them [27,40]. This ensures that participants who lack digital access or prefer nondigital interactions can still receive their results [40]. Participants who have different accessibility needs may be able to make use of existing digital adaptive technology [37] unless the researcher offers methods that support this intrinsically.

Secondly, text results may be adequate, though including relevant graphs, charts, and images can relay information more effectively [27,35,37,38,40,41,54]. Importantly, these must be in appropriate lay language, and educational materials must be provided to contextualize the results for participants [35,54,]. While participants are willing to receive favorable, neutral, and unfavorable results [37,38,40], excessive jargon or lack of clear directions of what can be done with the information impedes its use [36,38]. In many cases, participants will not act on neutral or positive information (such as clear screening results or standard contaminant levels) [38]. However, those who receive unfavorable information (such as indication of illness or other concern) express a clear desire to act on it and must be informed of who they can contact regarding their results, whether a research team member, their own personal HCP, or another expert for further information when results are unclear [36,38,40].

Thirdly, participants preferred receiving personalized information, while expecting many systems used methods to still maintain their privacy [35-37]. Articles emphasized the importance of deidentification whenever possible, which can involve using participant IDs or removing identifiers when storing and handling data up until the point of result dissemination, which can be further blinded from researchers by the system. This approach ensures that researchers do not have access to identifiable information during the result-creation process. Identifiers should only be used at the dissemination point to accurately link the results back to the participants, preserving their privacy and minimizing potential misuse of personalized data [35-37].

### Strengths and Limitations

This review surveyed articles across various research disciplines using a registered protocol. A reference librarian aided in developing and validating a comprehensive search strategy and methodology to avoid bias toward a specific research discipline.

However, there are also several limitations. Returning results is rarely the primary focus of research articles and is a method, not an intervention. As there is no scalable method for screening the body text of published work, this review was limited to articles where the method of returning results was reported in the abstract or title of the article. Similarly, the return of results is referred to using different terminology between research disciplines, so it is possible that instances of returning results were missed as the specific terminology was not included in the search strategy; this may bias our results, due to the authors’ greater familiarity with the terminology in some research fields rather than others. This may have also resulted in over-representing particular disciplines, primarily through iterative bibliography screening. In addition, this review did not consider any non-English language publications or publications after May 2023, which may have missed global or more contemporary research.

### Future Work

Future research should explore returning results to healthy participants or less vulnerable groups to explore the influence of altruism and the benefit to all research participants rather than only those who have a clear benefit to participating.

A generalizable results-sharing system should have a discipline-independent design available to all researchers. Many systems are highly tailored to a specific data and discipline type and, therefore, are unavailable to other researchers [27,35,37-40]. While this may contradict the requirement to provide educational and contextualizing materials, current solutions are too context-specific to be broadly adopted by other disciplines where participants could also benefit from receiving results. Consequently, this impedes their ability to scale and generalize, which can be addressed in future system design.

In addition, while most studies describe the methods used to return results, few include evaluation data to assess their effectiveness. Future research should incorporate evaluation components to determine whether these methods achieve their intended goals, such as increasing participant knowledge, reducing anxiety, or improving engagement with research findings. Moreover, none of the included studies compared different modalities for returning results, as they each focused on a single system design. Future work should explore participant preferences for different return methods to inform more user-centred approaches.

### Conclusions

This review identified 7 systems used to return individual results to research participants. While the return of results systems and methods are often tailored to the needs of the participants and the research discipline, some factors are universal. There is a clear need to support multiple dissemination methods, such as providing results digitally via a portal and (if requested) in physical form to allow equal opportunity for participants to receive and share their results. The inclusion of additional educational materials is crucial. Text is the most standard data format for results, though other, more creative formats can be well received by participants when applicable. Future work should include the development and testing of a discipline-independent research results sharing system.

## Supplementary material

10.2196/65606 Multimedia Appendix 1 Search strategy for the narrative review.

10.2196/65606 Multimedia Appendix 2 Data extraction form for narrative review.

10.2196/65606 Multimedia Appendix 3 Table of Supplementary Articles included in the narrative review.

10.2196/65606 Multimedia Appendix 4 Risk of Bias analysis.

10.2196/65606 Checklist 1 PRISMA Checklist

## Abbreviations

HCP: health care provider
PRISMA: Preferred Reporting Items for Systematic reviews and Meta-Analyses
