# Supplementary material for: Developing Requirements for a Standardized System to Return Individual Research Results Back to Study Participants: Narrative Review
Source: Interact J Med Res. 2025 Aug 18;14:e65606. doi: 10.2196/65606 (PMC12387377; doi:10.2196/65606)
Supplement: Multimedia Appendix 1 [file ijmr-v14-e65606-s001.docx]

| **#** | **Query** | **Results from 2023-05-15** |
| --- | --- | --- |
| 1 | TI=(research*) OR AB=(research*) OR AK=(research*) | 5,068,018 |
| 2 | TI=((“return” or “returning” or “returned” or “disseminating” or “dissemination” or “disseminated” or “share” or “shared” or “sharing”) NEAR/2 (“individual” or “individuals” or “individualized” or “personal” or “personalized” or “patient” or “patients” or “subject” or “subjects” or “participant” or “participants” or “research” or “community” or “communities”) NEAR/2 (“result” or “results” or “data”)) | 633 |
| 3 | AB =((“return” or “returning” or “returned” or “disseminating” or “dissemination” or “disseminated” or “share” or “shared” or “sharing”) NEAR/2 (“individual” or “individuals” or “individualized” or “personal” or “personalized” or “patient” or “patients” or “subject” or “subjects” or “participant” or “participants” or “research” or “community” or “communities”) NEAR/2 (“result” or “results” or “data”)) | 4,366 |
| 4 | AK =((“return” or “returning” or “returned” or “disseminating” or “dissemination” or “disseminated” or “share” or “shared” or “sharing”) NEAR/2 (“individual” or “individuals” or “individualized” or “personal” or “personalized” or “patient” or “patients” or “subject” or “subjects” or “participant” or “participants” or “research” or “community” or “communities”) NEAR/2 (“result” or “results” or “data”)) | 64 |
| 5 | #1 AND (#2 OR #3 OR #4) | 2,814 |
| 6 | PY = 2013-2023 | 30,361,752 |
| 7 | #5 AND #6 | 2,220 |

| **#** | **Query** | **Results from 2023-05-15** |
| --- | --- | --- |
| 1 | research.ti,tw,kf. | 2,042,434 |
| 2 | (("return" or "returning" or "returned" or "disseminating" or "dissemination" or "disseminated" or "share" or "shared" or "sharing") adj2 ("individual" or "individuals" or "individualized" or "personal" or "personalized" or "patient" or "patients" or "subject" or "subjects" or "participant" or "participants" or "research" or "community" or "communities") adj2 ("result" or "results" or "data")).ti,tw,kf. | 1807 |
| 3 | 1 and 2 | 1079 |
| 4 | limit 3 to yr="2013 - 2023" | 833 |
