# Supplementary material for: Developing Requirements for a Standardized System to Return Individual Research Results Back to Study Participants: Narrative Review
Source: Interact J Med Res. 2025 Aug 18;14:e65606. doi: 10.2196/65606 (PMC12387377; doi:10.2196/65606)
Supplement: Multimedia Appendix 2 [file ijmr-v14-e65606-s002.docx]

| **Extractor initials** |  |
| --- | --- |
| **Study Details** | |
| Title |  |
| Study Domain *(Healthcare, Education, Environmental Sciences, etc.)* |  |
| Author(s) |  |
| Country/Region |  |
| Year of publication |  |
| Full citation |  |
| DOI link |  |
| **Study characteristics** | |
| Study design |  |
| Study duration |  |
| **Population characteristics** | |
| Setting (*Academic, Commercial, Citizen Science, etc.*) |  |
| Location |  |
| Method of recruitment |  |
| Healthy participants *(yes/no)* |  |
| **Methodology** | |
| Aim of study |  |
| Start date |  |
| End date |  |
| Duration of participation in study |  |
| Risk of bias assessment |  |
| Design methods *(co-design, participatory design, etc.)* |  |
| **Participants** | |
| Total number |  |
| Age of Participants |  |
| Sex (*percentage female/male*) |  |
| Gender |  |
| Ethnicity/Race |  |
| **Data Characteristics** | |
| Data collection requirements *(authentication, storage, display, access, etc.)* |  |
| Information distribution requirements *(authentication, storage, upload, access, etc.)* |  |
| Variables shared |  |
| Presentation of information *(text, visualization, in-person, meeting, etc.)* |  |
| Mechanism of delivery *(text message, email, paper, etc.)* |  |
| Contextualization of results *(population, study cohort, reference ranges, no context)* |  |
| Identifiable *(aggregate vs non-identifiable vs personally/self identifiable data)* |  |
| Education of Results *(yes/no)* |  |
| **Other Information** | |
| Key conclusions of study authors (*including recommendations*) |  |
| Study limitations (*e.g., sample size, scalability, level of implementation, technical limitations, etc.*) |  |
