# Supplementary material for: Developing Requirements for a Standardized System to Return Individual Research Results Back to Study Participants: Narrative Review
Source: Interact J Med Res. 2025 Aug 18;14:e65606. doi: 10.2196/65606 (PMC12387377; doi:10.2196/65606)
Supplement: Multimedia Appendix 3 [file ijmr-v14-e65606-s003.docx]

| **Study** | **Participant Characteristics** | **Dissemination Technique** | **Information Format** | **Expert** | **Data Characteristics** |
| --- | --- | --- | --- | --- | --- |
| **Agongo, 2021**^1^ | 40-60 years in Kassena-Nankana districts (Ghana) | In Person | Verbal | Clinician | Aggregate, Personalized, Education |
| **Aktan-Collan, 2001**^2^ | Healthy family members of HNPCC families | Letter, In Person | Verbal | Genetic Counsellor | Personalized, Education |
| **Aktan-Collan, 2007**^3^ | Families with HNPCC | Unclear | Unclear | Genetic Counsellor | Personalized, Education |
| **Albada, 2015**^4^ | 18+ years who are first in their family to seek breast cancer genetic counseling | Letter, In Person | Text, Verbal | Clinician, Genetic Counsellor | Personalized |
| **Altman, 2008**^5^ | Region with elevated rates of breast cancer | Email, Phone | Text, Verbal, Graph | Other | Aggregate, Personalized, Education |
| **Anderson, 2014**^6^ | Hutterites | Phone, Letter, In Person | Text, Verbal | Genetic Counsellor | Personalized, Education |
| **Aspinwall, 2008**^7^ | Adults from melanoma pedigrees | Unclear | Verbal | Genetic Counsellor | Personalized, Education |
| **Beil, 2021**^8^ | Patients with aortic disease | Letter, In Person | Text, Verbal | Genetic Counsellor | Personalized, Education |
| **Beran, 2008**^9^ | Women who sought BRCA1/2 testing | Unclear | Verbal | Genetic Counsellor | Personalized, Education |
| **Bergenmar, 2009**^10^ | Families where germline CDKN2A mutations had been identified | In Person | Verbal | Clinician | Personalized, Education |
| **Bernardt, 2021**^11^ | Vulnerable patients (fragile socio-economic position, low health or digital literacy, mental illness, disability and/or a chronic medical condition | Portal | Unclear |  | Personalized |
| **Best, 2023**^12^ | Asthma | Phone, In Person, Other | Text, Verbal, Other |  | Aggregate, Personalized, Education |
| **Biesecker, 2018**^13^ | Healthy adults | Portal | Text, Verbal, Other | Genetic Counsellor | Personalized, Education |
| **Bonilla, 2022**^14^ | Latina breast cancer survivors | Letter | Text, Graph |  | Aggregate, Education |
| **Boronow, 2017**^15^ |  | Letter, Portal, In Person | Text, Verbal, Graph |  | Aggregate, Non-Identifiable, Education |
| **Bradbury, 2018**^16^ | BRCA1/2-negative women with early-onset breast cancer, multiple primary cancers, or a family history of breast cancer | Phone, In Person | Verbal | Genetic Counsellor | Not Reported |
| **Brannen, 2002**^17^ | Children (11 years) | In Person | Other |  | Aggregate |
| **Brown, 2017**^18^ | Intermediate coronary heart disease risk | Unclear | Verbal, Graph | Clinician, Genetic Counsellor | Aggregate, Personalized, Education |
| **Buck, 2010**^19^ | Women preconception to pregnancy or 12 menstrual cycles without pregnancy | Letter | Text |  | Aggregate, Personalized, Education |
| **Christensen, 2011**^20^ | Adults who had melanoma previously identified | Phone | Text, Verbal | Genetic Counsellor | Personalized, Education |
| **Claudio, 2018**^21^ | Low-income minority girls | Unclear | Text, Graph |  | Aggregate, Education |
| **Cope, 2023**^22^ | Babies of birthing parents at least 11 years of age | Email, Portal | Text, Verbal | Genetic Counsellor | Personalized, Education |
| **Crosslin, 2015**^23^ | Aged 50−65 years old, self-reported Asian or African ancestry and have EHR evidence of malignant hyperthermia and long QT syndrome were prioritized. Excluded if ever prescribed carbamazepine or had a current regimen of warfarin. | Unclear | Unclear |  | Personalized |
| **Culhane-Pera, 2017**^24^ | Hmong adults | In Person | Verbal | Other | Aggregate, Education |
| **Davis, 2014**^25^ | Turkish male with non-syndromic skeletal dysplasia | Unclear | Text, Verbal | Genetic Counsellor | Personalized, Education |
| **Dixon-Woods, 2011**^26^ | Mothers prescribed antibiotics during pregnancy | Letter | Unclear |  | Not Reported |
| **Dorval, 2005**^27^ | Women with inconclusive BRCA1/2 result | In Person | Verbal | Genetic Counsellor | Personalized, Education |
| **Eckert, 2006**^28^ | Adult children of living or deceased individual diagnosed with late-onset Alzheimer's disease | In Person | Text, Verbal, Graph | Genetic Counsellor | Aggregate, Personalized, Education |
| **Egli, 2019**^29^ | Children, 8-13 yr old | Unclear | Text, Verbal, Graph, Other |  | Aggregate, Personalized, Education |
| **Ellingson, 2015**^30^ | Patients age 18+ with newly diagnosed stage I-II breast cancer | Phone, In Person | Verbal | Genetic Counsellor | Personalized, Education |
| **Emmet, 2006a**^31^ | Residents near fluoropolymer production facility | Unclear | Unclear | Clinician | Personalized, Education |
| **Emmet, 2006b**^32^ | Previous diagnosis of liver or thyroid disease | Phone | Verbal | Clinician | Personalized, Education |
| **Exley, 2015**^33^ | Children and their mothers | Letter | Text |  | Aggregate, Personalized, Education |
| **Finlay, 2008**^34^ | Members of family with known BRCA1/2 mutations | Phone, Letter | Text, Verbal |  | Personalized, Education |
| **Fisher, 2016**^35^ | Orphan retinal degenerative diseases | Portal | Text, Graph, Other |  | Aggregate, Personalized |
| **Fuse, 2019**^36^ | People who suffered from the Great East Japan Earthquake | In Person | Verbal | Genetic Counsellor | Not Reported |
| **Galvin, 2020**^37^ | Older adults enrolled in cross-sectional clinical research | In Person | Text, Verbal | Clinician, Other | Personalized, Education |
| **Giannini, 2018**^38^ | Girls transitioning through puberty and persons living within five miles of a uranium refinery | Letter | Unclear |  | Aggregate, Personalized, Education |
| **Gilbert, 2022**^39^ | Patients with Inherited Retina Disease | Portal | Text, Other |  | Personalized |
| **Glanz, 2013**^40^ | Family history of melanoma | In Person | Text, Verbal | Genetic Counsellor | Personalized, Education |
| **Gordon, 2012**^41^ | Anyone that is at least 18 years old, have a valid email address, attend an in person informed consent session to consent to participate in the study, and provide a saliva sample for genomic analysis | Portal | Text, Other |  | Personalized, Education |
| **Graves, 2014**^42^ | Patients that have taken MMR genetic test | Phone, Letter | Text, Verbal | Genetic Counsellor | Personalized, Education |
| **Graves, 2013**^43^ | Adults 40+ years old | Phone, In Person | Text, Verbal, Graph | Genetic Counsellor | Aggregate, Personalized, Education |
| **Green, 2005**^44^ | Women who had been referred to a genetic counselor for evaluation of breast cancer risk | In Person | Verbal | Clinician, Genetic Counsellor | Personalized, Education |
| **Green, 2009**^45^ | Adult children of a living or deceased parent with Alzheimer’s disease | Unclear | Graph |  | Aggregate, Personalized |
| **Gritz, 2005**^46^ | People who have participated in concurrent studies involving hereditary nonpolyposis colorectal cancer genetic testing and psychosocial questionnaires | Phone, Letter | Verbal | Genetic Counsellor | Personalized, Education |
| **Haga, 2014**^47^ | Non-diabetic | Portal, In Person | Text, Verbal, Graph | Genetic Counsellor | Aggregate, Personalized |
| **Haines, 2011**^48^ | Women in the first trimester of pregnancy | Unclear | Unclear |  | Personalized |
| **Hamajima, 2004**^49^ | Smokers | In Person | Unclear |  | Not Reported |
| **Hartz, 2015**^50^ | Nicotine-dependent smokers from the St. Louis metropolitan area, minority population at high risk for depression and anxiety | In Person | Verbal, Other | Genetic Counsellor | Non-Identifiable, Education |
| **Hernick, 2011**^51^ | Girls 6–8 years of age | In Person | Text, Graph |  | Aggregate, Personalized, Education |
| **Horiuchi, 2021**^52^ | Japanese patients who had undergone surgical resection for cancer | Unclear | Unclear | Genetic Counsellor | Not Reported |
| **Huebner, 2023**^53^ | Metastatic breast cancer patients | Unclear | Unclear | Clinician | Not Reported |
| **Hughes, 2002**^54^ | Adult women who received BRCA1/2 test results | Unclear | Verbal | Genetic Counsellor | Personalized, Education |
| **Hutchison, 2021**^55^ | Behavioural health providers | Other | Verbal |  | Aggregate |
| **Ito, 2006**^56^ | Smokers | Letter, In Person | Verbal, Graph |  | Personalized, Education |
| **Jenkins, 2007**^57^ | Individuals undergoing BRCA1/2 predisposition genetic testing | Phone, Letter, In Person | Verbal | Clinician | Personalized, Education |
| **Johns, 2017**^58^ | Pancreatic cancer patients | Letter | Unclear | Clinician | Personalized, Education |
| **Johns, 2014**^59^ | Pancreatic cancer patients | Phone, Letter, In Person | Text, Verbal | Clinician, Other | Personalized, Education |
| **Judge, 2016**^60^ | Residents of the Ohio River Valley have been exposed to a toxic chemical, perfluorooctanoic acid | Unclear | Unclear |  | Aggregate, Personalized |
| **Kaphingst, 2012**^61^ | Groups traditionally underrepresented in genetics research (i.e., men, African Americans, and those with lower educational attainment) were oversampled | Phone, Letter | Text, Verbal |  | Personalized, Education |
| **Kasparian, 2009**^62^ | Individuals with a strong family history of melanoma and a known family-specific CDKN2A mutation | Unclear | Verbal | Genetic Counsellor | Unclear |
| **Kawame, 2022**^63^ | Sequenced genome cohort study involving healthy individuals affected by the Great East Japan Earthquake in 2011 | In Person | Verbal | Genetic Counsellor | Personalized, Education |
| **Keller, 2010**^64^ | Patients with breast and prostate cancer, chronic disease cohort which consists of participants with congestive heart failure | Portal, In Person | Text, Graph, Other |  | Personalized, Education |
| **Kelly, 2004**^65^ | Participants that were of Ashkenazi Jewish descent and had a personal or family history indicating risk for a BRCA1/2 mutation | Phone | Verbal | Genetic Counsellor | Personalized, Education |
| **Keogh, 2014**^66^ | Cases of colorectal cancer at any age | Phone, Letter, In Person | Text, Verbal | Clinician | Personalized, Education |
| **Kinney, 2005**^67^ | African-American with BRCA1 mutation | Unclear | Verbal | Genetic Counsellor | Personalized, Education |
| **Kullo, 2018**^68^ | Participants who had hypercholesterolemia, colon polyps | Phone, Letter, In Person | Text, Verbal | Genetic Counsellor | Personalized, Education |
| **Kurian, 2014**^69^ | Patients previously referred for BCRA1/2 testing | Phone, In Person | Verbal | Genetic Counsellor | Personalized, Education |
| **LaRusse, 2005**^70^ | Women who received a lifetime risk estimate based on family history and gender, as well as women who possessed an APOE genotype and received a lifetime risk estimate based on genotype, gender, and family history | In Person | Text, Verbal, Other | Genetic Counsellor | Personalized, Education |
| **Leitsalu, 2016**^71^ | Estonian biobank cohort | Letter, In Person | Text, Verbal | Genetic Counsellor | Personalized, Education |
| **Leitsalu, 2021**^72^ | Estonian biobank cohort | Unclear | Text | Clinician, Genetic Counsellor | Personalized, Education |
| **Leitsalu, 2022**^73^ | Estonian biobank cohort | In Person | Text, Verbal | Genetic Counsellor | Personalized, Education |
| **Lemke, 2018**^74^ | NorthShore adult patients | Other | Unclear |  | Personalized |
| **Lerman, 1996**^75^ | Adult male and female members of families with BRCA 1 linked hereditary breast-ovarian cancer | Phone, In Person | Verbal | Genetic Counsellor | Personalized, Education |
| **Lewis, 2018**^76^ | Post reproductive-aged participants in the ClinSeq cohort | Portal | Verbal | Genetic Counsellor | Personalized, Education |
| **Lipkus, 2004**^77^ | African American smokers | Phone, Letter | Text, Verbal, Graph | Genetic Counsellor | Aggregate, Personalized, Education |
| **Llewellyn, 2015**^78^ | 70% participants with a chronic illness, and 30% healthy | Phone, Letter | Text, Verbal, Graph | Other | Personalized, Education |
| **Llewellyn, 2016**^79^ | 70% participants with a chronic illness, and 30% healthy | Phone | Text, Verbal, Graph | Other | Personalized, Education |
| **Lodder, 2001**^80^ | Women with a 25 or 50% risk of being a BRCA1/BRCA2 mutation carrier who applied for BRCA1 genetic testing | Unclear | Verbal | Genetic Counsellor | Personalized, Education |
| **Lynch, 1993**^81^ | High-risk members of the hereditary breast/ ovarian cancer syndrome kindred | Phone | Verbal | Clinician, Genetic Counsellor, Other | Personalized, Education |
| **Lynch, 2020**^82^ | Participants already enrolled at eMERGE Clinical sites | Phone, Letter, Portal | Text |  | Personalized, Education |
| **Maeda, 2020**^83^ | All residents older than 20 years of age who live in Iwaki district of Hirosaki city, Japan | Unclear | Verbal | Genetic Counsellor | Personalized, Education |
| **Marteau, 2005**^84^ | Adult children of a living or deceased person with AD | Unclear | Verbal | Genetic Counsellor | Personalized, Education |
| **McBride, 2000**^85^ | Smokers | Phone, Letter, In Person | Text, Verbal, Other | Expert | Personalized, Education |
| **McBride, 2002**^86^ | African American smokers | Phone, Letter | Text, Verbal, Graph | Genetic Counsellor | Personalized, Education |
| **Meagher, 2021**^87^ | Biobank participants who were notified by mail of an individual research result indicating increased risk for adverse events associated with the common cancer drug 5-fluorouracil | Letter | Text |  | Personalized, Education |
| **Monnard, 2021**^88^ | Communities comprised predominantly people of color and had median household incomes, high school graduation rates, and employment rates lower than national averages | In Person, Other | Text, Verbal, Graph, Other | Other | Aggregate, Education |
| **Muse, 2021**^89^ | Adults with a smartphone and an existing 23andMe genetic profiling | Portal | Text, Graph |  | Personalized, Education |
| **Ndase, 2014**^90^ | African heterosexual HIV serodiscordant couples | In Person, Other | Text, Verbal | Other | Aggregate |
| **Nestor, 2020**^91^ | Nephrology patients | Phone, In Person | Text, Verbal | Clinician | Personalized |
| **Nickerson, 2015**^92^ | Overweight/obese and prediabetic by oral glucose tolerance testing | In Person | Unclear |  | Personalized |
| **Norelli, 2021**^93^ | Men who have sex with men | Unclear | Unclear | Other | Personalized |
| **Nusbaum, 2013**^94^ | Male and female primary care patients aged 40 and older | In Person | Text, Verbal, Graph | Genetic Counsellor | Personalized, Education |
| **Oerlemans, 2017**^95^ | Patients with Hodgkin lymphoma and non-Hodgkin lymphoma | Unclear | Graph |  | Aggregate |
| **Ohneda, 2022**^96^ | Population-based genome cohort | Phone, Letter | Text, Verbal | Genetic Counsellor | Personalized, Education |
| **Ohneda, 2023**^97^ | BRCA1/2 pathogenic variant carriers | In Person | Verbal | Genetic Counsellor | Personalized, Education |
| **Olson, 2017**^98^ | Selected from population-based cohort | Letter, Portal | Text |  | Personalized, Education |
| **Ondenge, 2015**^99^ | Breastfeeding individuals in Kisumu | In Person | Text, Verbal, Other |  | Aggregate |
| **Ottman, 2018**^100^ | Participants already enrolled in genetic research in the epilepsies | In Person | Verbal | Genetic Counsellor | Personalized, Education |
| **Patrick-Miller, 2013**^101^ | Patients presenting for clinical BRCA1/2 testing who were over 18 years old | Phone, Letter, In Person | Verbal | Genetic Counsellor | Personalized, Education |
| **Peltekova, 2021**^102^ | Children participants in large genomics studies in Autism Spectrum Disorders | Unclear | Unclear | Clinician, Genetic Counsellor | Personalized |
| **Perovich, 2018**^103^ | Children with doctor-diagnosed asthma | Letter, In Person | Text, Graph, Other |  | Aggregate, Personalized, Education |
| **Plon, 2000**^104^ | Adult men and women of at least 50% Ashkenazi ancestry | Letter | Unclear |  | Personalized, Education |
| **Polka, 2021**^105^ | Homes in two environmental justice communities, children in areas with manganese in their local drinking water | Letter | Text, Graph |  | Personalized, Education |
| **Pulford, 2016**^106^ | Women with FIGO Stage II-IV epithelial ovarian, fallopian tube, or primary peritoneal cancer who had not progressed after first-line chemotherapy | Unclear | Unclear | Other | Aggregate, Education |
| **Quandt, 2004**^107^ | Homes of Latino/a migrant and seasonal farmworkers | In Person | Verbal, Graph | Other | Aggregate, Personalized, Education |
| **Ramirez-Vazquez, 2019**^108^ | Participants from Albacete | Unclear | Unclear |  | Personalized, Education |
| **Richards, 1997**^109^ | Ashkenazic Jewish adults 21+ years | Phone, Letter, In Person | Text, Verbal | Genetic Counsellor | Personalized, Education |
| **Rohlman, 2019**^110^ | Swinomish Indian Tribal Community (previously impacted by an air toxic release from one of two nearby oil refineries) | Unclear | Text, Graph |  | Aggregate, Personalized, Education |
| **Rowley, 2019**^111^ | Healthy Australian women | Phone, Letter | Verbal | Genetic Counsellor | Personalized, Education |
| **Salvi, 2022**^112^ | India; children 9 months to < 5 years; 5 to < 15 years of age, and women 15 to < 50 years of age before and after the measles and rubella (MR) vaccination campaigns | In Person | Text, Verbal | Clinician, Other | Personalized, Education |
| **Samaan, 2013**^113^ | Men and women ≥ 30 years of age of South Asian ancestry | Portal | Text, Graph |  | Personalized, Education |
| **Sanderson, 2009**^114^ | Patients with stage IIIB/IV lung cancer | Portal | Text, Graph, Other |  | Personalized, Education |
| **Sandhaus, 2019**^115^ | Gardeners living near hazardous waste or resource extraction activities | Email, Letter, In Person | Text, Verbal, Graph | Other | Aggregate, Personalized, Education |
| **Savatt, 2018**^116^ | Anyone who has had genetic testing regardless of genetic test results or diagnosis | Email, Portal | Unclear | Other | Not Reported |
| **Schollaert, 2021**^117^ | Chelsea residents | Letter, In Person | Text, Verbal | Other | Aggregate, Personalized, Education |
| **Schwartz, 2014**^118^ | Women aged 21 to 85 years who did not have newly diagnosed or metastatic cancer | Phone, Letter, In Person | Verbal, Other | Genetic Counsellor | Personalized |
| **Shaibi, 2020**^119^ | Low-income Latino | Phone, Letter, In Person | Text, Verbal | Genetic Counsellor | Personalized, Education |
| **Sharff, 2012**^120^ | Mothers who underwent genetic counseling and testing for BRCA1/2 and their untested spouses or partners | Unclear | Verbal | Genetic Counsellor | Personalized |
| **Shepherd, 2021**^121^ | Women recruited from government-funded breast cancer screening in the general population | Phone, Letter | Verbal | Genetic Counsellor | Personalized, Education |
| **Siegfried, 2013**^122^ | Families with dilated cardiomyopathy of all sizes | Letter | Text |  | Personalized, Education |
| **Smith, 1999**^123^ | People identified with a BRCA1 mutation | In Person | Verbal | Genetic Counsellor | Personalized |
| **Sommer, 2020**^124^ | Male participants with ≤6 months of pregnancy attempt time at study enrolment, and whose female partners reported a regular menstrual cycle | Unclear | Unclear |  | Not Reported |
| **South, 2021**^125^ | Women with ovarian cancer | Email, Letter, Portal | Text, Other | Other | Personalized, Education |
| **Spagnolo, 2020**^126^ | Republic of Coˆte d’Ivoire (area near places with Ebola outbreak) | In Person | Text, Verbal | Other | Aggregate |
| **Stefansdottir, 2020**^127^ | Anyone that has already donated to biobank | Phone, Portal | Text, Verbal | Genetic Counsellor | Personalized, Aggregate |
| **Streeten, 2020**^128^ | Amish individuals | Letter, In Person | Text, Verbal | Genetic Counsellor | Personalized, Education |
| **Tabor, 2017**^129^ |  | Portal | Text |  | Personalized, Education |
| **Tomsho, 2018**^130^ |  | Letter | Other |  | Aggregate, Education |
| **Tzovaras, 2019**^131^ |  | Portal | Unclear |  | Not Reported |
| **van de Poll-Franse, 2022**^132^ | Cancer patients/survivors | Portal | Unclear |  | Aggregate, Personalized, Education |
| **van Dijk, 2005**^133^ | Women who received an uninformative BRCA1/2 test result | Unclear | Verbal | Genetic Counsellor | Personalized, Education |
| **van Oostrom, 2007**^134^ | Applicants for genetic susceptibility testing for a known familial pathogenic BRCA1/2 mutation or a mutation predisposing to HNPCC aged 18 years or older | Letter | Text, Verbal | Genetic Counsellor | Personalized, Education |
| **Vassy, 2014**^135^ | Patients from primary care and cardiology | Portal | Text | Clinician | Personalized, Educational |
| **von Behren, 2022**^136^ | People in homes still standing within the burn zone of the 2018 Camp Fire in California | In Person | Text, Verbal | Other | Aggregate, Personalized, Education |
| **Wakefield, 2013**^137^ | Multi-case breast and/or ovarian cancer families | Letter | Text |  | Education |
| **Widén, 2022**^138^ | Majority of participants recruited were overweight or obese | Portal | Text, Graph |  | Personalized, Education |
| **Wiggins, 1992**^139^ | Recruited from Canadian program of genetic testing to predict the risk of Huntington's disease | Unclear | Unclear |  | Personalized |
| **Williams, 2018**^140^ | Parents of children with undiagnosed Autism Spectrum Disorder, unexplained Intellectual Disability and/or multiple congenital anomalies | In Person | Text, Verbal | Clinician, Genetic Counsellor | Personalized, Education |

1. Agongo G, Debpuur C, Amenga-Etego L, et al. Community engagement and feedback of results in the H3Africa AWI-Gen project: Experiences from the Navrongo Demographic and Health Surveillance site in Northern Ghana. *AAS Open Res*. 2021;4(101740247):15. doi:10.12688/aasopenres.13081.1

2. Aktan-Collan K, Haukkala A, Mecklin JP, Uutela A, K��ri�inen H. Psychological consequences of predictive genetic testing for hereditary non-polyposis colorectal cancer (HNPCC): A prospective follow-up study. *Int J Cancer*. 2001;93(4):608-611. doi:10.1002/ijc.1372

3. Aktan-Collan K, Haukkala A, Pylvanainen K, et al. Direct contact in inviting high-risk members of hereditary colon cancer families to genetic counselling and DNA testing. *J Med Genet*. 2007;44(11):732-738. doi:10.1136/jmg.2007.051581

4. Albada A, Van Dulmen S, Spreeuwenberg P, Ausems MGEM. Follow-up effects of a tailored pre-counseling website with question prompt in breast cancer genetic counseling. *Patient Educ Couns*. 2015;98(1):69-76. doi:10.1016/j.pec.2014.10.005

5. Altman RG, Morello-Frosch R, Brody JG, Rudel R, Brown P, Averick M. Pollution Comes Home and Gets Personal: Women’s Experience of Household Chemical Exposure. *J Health Soc Behav*. 2008;49(4):417-435. doi:10.1177/002214650804900404

6. Anderson RL, Murray K, Chong JX, et al. Disclosure of Genetic Research Results to Members of a Founder Population. *J Genet Couns*. 2014;23(6):984-991. doi:10.1007/s10897-014-9721-8

7. Aspinwall LG, Leaf SL, Dola ER, Kohlmann W, Leachman SA. CDKN2A/p16 genetic test reporting improves early detection intentions and practices in high-risk melanoma families. *Cancer Epidemiol Biomark Prev Publ Am Assoc Cancer Res Cosponsored Am Soc Prev Oncol*. 2008;17(6):1510-1519. doi:10.1158/1055-9965.EPI-08-0010

8. Beil A, Hornsby W, Uhlmann WR, et al. Disclosure of clinically actionable genetic variants to thoracic aortic dissection biobank participants. *BMC Med Genomics*. 2021;14(1):66. doi:10.1186/s12920-021-00902-5

9. Beran TM, Stanton AL, Kwan L, et al. The Trajectory of Psychological Impact in BRCA1/2 Genetic Testing: Does Time Heal? *Ann Behav Med*. 2008;36(2):107-116. doi:10.1007/s12160-008-9060-9

10. Bergenmar M, Hansson J, Brandberg Y. Family members’ perceptions of genetic testing for malignant melanoma – A prospective interview study. *Eur J Oncol Nurs*. 2009;13(2):74-80. doi:10.1016/j.ejon.2008.12.003

11. Bernaerdt J, Moerenhout T, Devisch I. Vulnerable patients’ attitudes towards sharing medical data and granular control in patient portal systems: an interview study. *J Eval Clin Pract*. 2021;27(2):429-437. doi:10.1111/jep.13465

12. Best LG, O’Leary M, O’Leary R, Lawrence W, Torgerson DG. Return of Participants’ Incidental Genetic Research Findings: Experience from a Case-Control Study of Asthma in an American Indian Community. *Res Sq*. 2023;(101768035). doi:10.21203/rs.3.rs-2485539/v1

13. Biesecker BB, Lewis KL, Umstead KL, et al. Web Platform vs In-Person Genetic Counselor for Return of Carrier Results From Exome Sequencing: A Randomized Clinical Trial. *JAMA Intern Med*. 2018;178(3):338. doi:10.1001/jamainternmed.2017.8049

14. Bonilla J, Alhomsi A, Santoyo-Olsson J, et al. Sharing research results with Latina breast cancer survivors who participated in a community-engaged behavioral RCT study: a descriptive cross-sectional survey study. *TRIALS*. 2022;23(1). doi:10.1186/s13063-021-05945-8

15. Boronow KE, Susmann HP, Gajos KZ, et al. DERBI: A Digital Method to Help Researchers Offer “Right-to-Know” Personal Exposure Results. *Environ Health Perspect*. 2017;125(2):A27-A33. doi:10.1289/EHP702

16. Bradbury A, Patrick-Miller L, Egleston B, et al. Returning Individual Genetic Research Results to Research Participants: Uptake and Outcomes Among Patients With Breast Cancer. *JCO Precis Oncol*. 2018;2:1-24. doi:10.1200/PO.17.00250

17. Brannen J. The use of video in research dissemination: Children as experts on their own family lives. *Int J Soc Res Methodol*. 2002;5(2):173-180. doi:10.1080/13645570110118700

18. Brown SAN, Jouni H, Marroush TS, Kullo IJ. Effect of Disclosing Genetic Risk for Coronary Heart Disease on Information Seeking and Sharing: The MI-GENES Study (Myocardial Infarction Genes). *Circ Cardiovasc Genet*. 2017;10(4):e001613. doi:10.1161/CIRCGENETICS.116.001613

19. Buck AJ, Vena JE, McGuinness BM, Cooney MA, Louis GM. RCesoeamrchmunicating serum chemical concentrations to study participants: follow up survey. Published online 2010.

20. Christensen KD, Roberts JS, Shalowitz DI, et al. Disclosing Individual CDKN2A Research Results to Melanoma Survivors: Interest, Impact, and Demands on Researchers. *Cancer Epidemiol Biomarkers Prev*. 2011;20(3):522-529. doi:10.1158/1055-9965.EPI-10-1045

21. Claudio L, Gilmore J, Roy M, Brenner B. Communicating environmental exposure results and health information in a community-based participatory research study. *BMC Public Health*. 2018;18(1):784. doi:10.1186/s12889-018-5721-1

22. Cope H, Lincoln-Boyea B, Gwaltney A, et al. Use of a web-based portal to return normal individual research results in Early Check: Exploring user behaviors and attitudes. *Clin Genet*. 2023;103(6):672-680. doi:10.1111/cge.14325

23. Crosslin DR, Robertson PD, Carrell DS, et al. Prospective participant selection and ranking to maximize actionable pharmacogenetic variants and discovery in the eMERGE Network. *Genome Med*. 2015;7(1):67. doi:10.1186/s13073-015-0181-z

24. Culhane-Pera KA, Straka RJ, Moua M, et al. Engaging Hmong adults in genomic and pharmacogenomic research: Toward reducing health disparities in genomic knowledge using a community-based participatory research approach. *J Community Genet*. 2017;8(2):117-125. doi:10.1007/s12687-017-0292-x

25. Davis DS. Alzheimer disease and pre-emptive suicide. *J Med Ethics*. 2014;40(8):543-549. doi:10.1136/medethics-2012-101022

26. Dixon-Woods M, Tarrant C, Jackson CJ, Jones DR, Kenyon S. Providing the results of research to participants: a mixed-method study of the benefits and challenges of a consultative approach. *Clin Trials*. 2011;8(3):330-341. doi:10.1177/1740774511403514

27. Dorval M, Gauthier G, Maunsell E, et al. No Evidence of False Reassurance among Women with an Inconclusive *BRCA1/2* Genetic Test Result. *Cancer Epidemiol Biomarkers Prev*. 2005;14(12):2862-2867. doi:10.1158/1055-9965.EPI-05-0512

28. Eckert SL, Katzen H, Roberts JS, et al. Recall of disclosed Apolipoprotein E genotype and lifetime risk estimate for Alzheimer’s disease: The REVEAL Study. *Genet Med*. 2006;8(12):746-751. doi:10.1097/01.gim.0000250197.44245.a3

29. Egli V, Carroll P, Donnellan N, Mackay L, Anderson B, Smith M. Disseminating research results to kids: practical tips from the Neighbourhoods for Active Kids study. *KOTUITUI-N Z J Soc Sci ONLINE*. 2019;14(2):257-275. doi:10.1080/1177083X.2019.1621909

30. Ellingson M, Hart S, Kalari K, et al. Exome sequencing reveals frequent deleterious germline variants in cancer susceptibility genes in women with invasive breast cancer undergoing neoadjuvant chemotherapy. *BREAST CANCER Res Treat*. 2015;153(2):435-443. doi:10.1007/s10549-015-3545-6

31. Emmett EA, Shofer FS, Zhang H, Freeman D, Desai C, Shaw LM. Community exposure to perfluorooctanoate: relationships between serum concentrations and exposure sources. *J Occup Environ Med*. 2006;48(8):759-770. doi:10.1097/01.jom.0000232486.07658.74

32. Emmett EA, Zhang H, Shofer FS, et al. Community exposure to perfluorooctanoate: relationships between serum levels and certain health parameters. *J Occup Environ Med*. 2006;48(8):771-779. doi:10.1097/01.jom.0000233380.13087.37

33. Exley K, Cano N, Aerts D, et al. Communication in a Human biomonitoring study: Focus group work, public engagement and lessons learnt in 17 European countries. *Environ Res*. 2015;141:31-41. doi:10.1016/j.envres.2014.12.003

34. Finlay E, Stopfer JE, Burlingame E, et al. Factors Determining Dissemination of Results and Uptake of Genetic Testing in Families with Known *BRCA1/2* Mutations. *Genet Test*. 2008;12(1):81-91. doi:10.1089/gte.2007.0037

35. Fisher JK, Bromley RL, Mansfield BC. My Retina Tracker^TM^: An On-line International Registry for People Affected with Inherited Orphan Retinal Degenerative Diseases and their Genetic Relatives - A New Resource. In: Bowes Rickman C, LaVail MM, Anderson RE, Grimm C, Hollyfield J, Ash J, eds. *Retinal Degenerative Diseases*. Vol 854. Advances in Experimental Medicine and Biology. Springer International Publishing; 2016:245-251. doi:10.1007/978-3-319-17121-0_33

36. Fuse N, Sakurai-Yageta M, Katsuoka F, et al. Establishment of Integrated Biobank for Precision Medicine and Personalized Healthcare: The Tohoku Medical Megabank Project. *JMA J*. 2019;2(2):113-122. doi:10.31662/jmaj.2019-0014

37. Galvin JE, Tolea MI, Chrisphonte S. What older adults do with the results of dementia screening programs. *PloS One*. 2020;15(7):e0235534. doi:10.1371/journal.pone.0235534

38. Giannini CM, Herrick RL, Buckholz JM, Daniels AR, Biro FM, Pinney SM. Comprehension and perceptions of study participants upon receiving perfluoroalkyl substance exposure biomarker results. *Int J Hyg Environ Health*. 2018;221(7):1040-1046. doi:10.1016/j.ijheh.2018.07.005

39. Gilbert RM, Sumodhee D, Pontikos N, et al. Collaborative Research and Development of a Novel, Patient-Centered Digital Platform (MyEyeSite) for Rare Inherited Retinal Disease Data: Acceptability and Feasibility Study. *JMIR Form Res*. 2022;6(1):e21341. doi:10.2196/21341

40. Glanz K, Volpicelli K, Kanetsky PA, et al. Melanoma Genetic Testing, Counseling, and Adherence to Skin Cancer Prevention and Detection Behaviors. *Cancer Epidemiol Biomarkers Prev*. 2013;22(4):607-614. doi:10.1158/1055-9965.EPI-12-1174

41. Gordon ES, Griffin G, Wawak L, Pang H, Gollust SE, Bernhardt BA. “It’s Not Like Judgment Day”: Public Understanding of and Reactions to Personalized Genomic Risk Information. *J Genet Couns*. 2012;21(3):423-432. doi:10.1007/s10897-011-9476-4

42. Graves K, Sinicrope P, Esplen M, et al. Communication of genetic test results to family and health-care providers following disclosure of research results. *Genet Med*. 2014;16(4):294-301. doi:10.1038/gim.2013.137

43. Graves KD, Leventhal KG, Nusbaum R, et al. Behavioral and psychosocial responses to genomic testing for colorectal cancer risk. *Genomics*. 2013;102(2):123-130. doi:10.1016/j.ygeno.2013.04.002

44. Green MJ, Peterson SK, Baker MW, et al. Use of an educational computer program before genetic counseling for breast cancer susceptibility: Effects on duration and content of counseling sessions. *Genet Med*. 2005;7(4):221-229. doi:10.1097/01.GIM.0000159905.13125.86

45. Green RC, Cupples LA, Whitehouse PJ, Eckert SL, Quaid KA, Farrer LA. Disclosure of APOE Genotype for Risk of Alzheimer’s Disease. *N Engl J Med*. Published online 2009.

46. Gritz ER, Peterson SK, Vernon SW, et al. Psychological Impact of Genetic Testing for Hereditary Nonpolyposis Colorectal Cancer. *J Clin Oncol*. 2005;23(9):1902-1910. doi:10.1200/JCO.2005.07.102

47. Haga SB, Barry WT, Mills R, et al. Impact of Delivery Models on Understanding Genomic Risk for Type 2 Diabetes. *Public Health Genomics*. 2014;17(2):95-104. doi:10.1159/000358413

48. Haines DA, Arbuckle TE, Lye E, et al. Reporting results of human biomonitoring of environmental chemicals to study participants: a comparison of approaches followed in two Canadian studies. *J Epidemiol Community Health*. 2011;65(3):191-198. doi:10.1136/jech.2008.085597

49. Hamajima N, Atsuta Y, Goto Y, Ito H. A pilot study on genotype announcement to induce smoking cessation by Japanese smokers. *Asian Pac J Cancer Prev APJCP*. 2004;5(4):409-413.

50. Hartz SM, Olfson E, Culverhouse R, et al. Return of individual genetic results in a high-risk sample: enthusiasm and positive behavioral change. *Genet Med*. 2015;17(5):374-379. doi:10.1038/gim.2014.110

51. Hernick AD, Brown MK, Pinney SM, Biro FM, Ball KM, Bornschein RL. Sharing Unexpected Biomarker Results with Study Participants. *Environ Health Perspect*. 2011;119(1):1-5. doi:10.1289/ehp.1001988

52. Horiuchi Y, Matsubayashi H, Kiyozumi Y, et al. Disclosure of secondary findings in exome sequencing of 2480 Japanese cancer patients. *Hum Genet*. 2021;140(2):321-331. doi:10.1007/s00439-020-02207-6

53. Huebner H, Ruebner M, Kurbacher C, et al. Return of individual genomic research results within the PRAEGNANT multicenter registry study. *BREAST CANCER Res Treat*. 2023;197(2):355-368. doi:10.1007/s10549-022-06795-x

54. Hughes C, Lerman C, Schwartz M, et al. All in the family: Evaluation of the process and content of sisters’ communication about *BRCA1* and *BRCA2* genetic test results. *Am J Med Genet*. 2002;107(2):143-150. doi:10.1002/ajmg.10110

55. Hutchison SL, Herschell AD, Clauss K, Hovorka K, Wasilchak DS, Hurford MO. Payer–Provider Partnership to Identify Successful Retention Strategies for the Behavioral Health Workforce. *Prog Community Health Partnersh Res Educ Action*. 2021;15(2):151-160. doi:10.1353/cpr.2021.0018

56. Ito H, Matsuo K, Wakai K, et al. An intervention study of smoking cessation with feedback on genetic cancer susceptibility in Japan. *Prev Med*. 2006;42(2):102-108. doi:10.1016/j.ypmed.2005.10.006

57. Jenkins J, Calzone KA, Dimond E, et al. Randomized comparison of phone versus in-person BRCA1/2 predisposition genetic test result disclosure counseling. *Genet Med*. 2007;9(8):487-495. doi:10.1097/GIM.0b013e31812e6220

58. Johns A, Mckay S, Humphris J, et al. Lost in translation: returning germline genetic results in genome-scale cancer research. *GENOME Med*. 2017;9. doi:10.1186/s13073-017-0430-4

59. Johns A, Miller D, Simpson S, et al. Returning individual research results for genome sequences of pancreatic cancer. *GENOME Med*. 2014;6. doi:10.1186/gm558

60. Judge JM, Brown P, Brody JG, Ryan S. The Exposure Experience: Ohio River Valley Residents Respond to Local Perfluorooctanoic Acid (PFOA) Contamination. *J Health Soc Behav*. 2016;57(3):333-350. doi:10.1177/0022146516661595

61. Kaphingst KA, McBride CM, Wade C, et al. Patients’ understanding of and responses to multiplex genetic susceptibility test results. *Genet Med*. 2012;14(7):681-687. doi:10.1038/gim.2012.22

62. Kasparian NA, Meiser B, Butow PN, Simpson JM, Mann GJ. Genetic testing for melanoma risk: a prospective cohort study of uptake and outcomes among Australian families. *Genet Med*. 2009;11(4):265-278. doi:10.1097/GIM.0b013e3181993175

63. Kawame H, Fukushima A, Fuse N, et al. The return of individual genomic results to research participants: design and pilot study of Tohoku Medical Megabank Project. *J Hum Genet*. 2022;67(1):9-17. doi:10.1038/s10038-021-00952-8

64. Keller MA, Gordon ES, Stack CB, et al. Coriell Personalized Medicine Collaborative ^®^ : A Prospective Study of the Utility Of Personalized Medicine. *Pers Med*. 2010;7(3):301-317. doi:10.2217/pme.10.13

65. Kelly K, Leventhal H, Marvin M, Toppmeyer D, Baran J, Schwalb M. Cancer Genetics Knowledge and Beliefs and Receipt of Results in Ashkenazi Jewish Individuals Receiving Counseling for BRCA1/2 Mutations. *Cancer Control*. 2004;11(4):236-244. doi:10.1177/107327480401100405

66. Keogh LA, Fisher D, Sheinfeld Gorin S, et al. How do researchers manage genetic results in practice? The experience of the multinational Colon Cancer Family Registry. *J Community Genet*. 2014;5(2):99-108. doi:10.1007/s12687-013-0148-y

67. Kinney AY, Bloor LE, Mandal D, et al. The impact of receiving genetic test results on general and cancer‐specific psychologic distress among members of an African‐American kindred with a *BRCA1* mutation. *Cancer*. 2005;104(11):2508-2516. doi:10.1002/cncr.21479

68. Kullo IJ, Olson J, Fan X, et al. The Return of Actionable Variants Empirical (RAVE) Study, a Mayo Clinic Genomic Medicine Implementation Study: Design and Initial Results. *Mayo Clin Proc*. 2018;93(11):1600-1610. doi:10.1016/j.mayocp.2018.06.026

69. Kurian AW, Hare EE, Mills MA, et al. Clinical Evaluation of a Multiple-Gene Sequencing Panel for Hereditary Cancer Risk Assessment. *J Clin Oncol*. 2014;32(19):2001-2009. doi:10.1200/JCO.2013.53.6607

70. Larusse S, Roberts JS, Marteau TM, et al. Genetic susceptibility testing versus family history–based risk assessment: Impact on perceived risk of Alzheimer disease. *Genet Med*. 2005;7(1):48-53. doi:10.1097/01.GIM.0000151157.13716.6C

71. Leitsalu L, Alavere H, Jacquemont S, et al. Reporting incidental findings of genomic disorder-associated copy number variants to unselected biobank participants. *Pers Med*. 2016;13(4):303-314. doi:10.2217/pme-2016-0009

72. Leitsalu L, Palover M, Sikka TT, et al. Genotype-first approach to the detection of hereditary breast and ovarian cancer risk, and effects of risk disclosure to biobank participants. *Eur J Hum Genet*. 2021;29(3):471-481. doi:10.1038/s41431-020-00760-2

73. Leitsalu L, Reigo A, Palover M, et al. Lessons learned during the process of reporting individual genomic results to participants of a population-based biobank. *Eur J Hum Genet EJHG*. 2022;(9302235, b4k). doi:10.1038/s41431-022-01196-6

74. Lemke AA, Hulick PJ, Wake DT, et al. Patient Perspectives Following Pharmacogenomics Results Disclosure in an Integrated Health System. *Pharmacogenomics*. 2018;19(4):321-331. doi:10.2217/pgs-2017-0191

75. Lerman C. BRCA1 testing in families with hereditary breast-ovarian cancer. A prospective study of patient decision making and outcomes. *JAMA J Am Med Assoc*. 1996;275(24):1885-1892. doi:10.1001/jama.275.24.1885

76. Lewis KL, Umstead KL, Johnston JJ, et al. Outcomes of Counseling after Education about Carrier Results: A Randomized Controlled Trial. *Am J Hum Genet*. 2018;102(4):540-546. doi:10.1016/j.ajhg.2018.02.009

77. Lipkus IM, McBride CM, Pollak KI, Lyna P, Bepler G. Interpretation of Genetic Risk Feedback Among African American Smokers With Low Socioeconomic Status. *Health Psychol*. 2004;23(2):178-188. doi:10.1037/0278-6133.23.2.178

78. Llewellyn AM, Skevington SM. Using guided individualised feedback to review self-reported quality of life in health and its importance. *Psychol Health*. 2015;30(3):301-317. doi:10.1080/08870446.2014.972396

79. Llewellyn AM, Skevington SM. Evaluating a new methodology for providing individualized feedback in healthcare on quality of life and its importance, using the WHOQOL-BREF in a community population. *Qual Life Res*. 2016;25(3):605-614. doi:10.1007/s11136-015-1132-2

80. Lodder L, Frets PG, Trijsburg RW, et al. Psychological impact of receiving aBRCA1/BRCA2 test result. *Am J Med Genet*. 2001;98(1):15-24. doi:10.1002/1096-8628(20010101)98:1<15::AID-AJMG1014>3.0.CO;2-0

81. Lynch HT, Watson P, Conway TA, et al. DNA screening for breast/ovarian cancer susceptibility based on linked markers. A family study. *Arch Intern Med*. 1993;153(17):1979-1987.

82. Lynch J, Sharp R, Aufox S, et al. Understanding the Return of Genomic Sequencing Results Process: Content Review of Participant Summary Letters in the eMERGE Research Network. *J Pers Med*. 2020;10(2):38. doi:10.3390/jpm10020038

83. Maeda Y, Sasaki A, Kasai S, et al. Prevalence of the mitochondrial 1555 A>G and 1494 C>T mutations in a community-dwelling population in Japan. *Hum Genome Var*. 2020;7(1):27. doi:10.1038/s41439-020-00115-9

84. Marteau TM, Roberts S, LaRusse S, Green RC. Predictive Genetic Testing for Alzheimer’s Disease: Impact upon Risk Perception. *Risk Anal*. 2005;25(2):397-404. doi:10.1111/j.1539-6924.2005.00598.x

85. McBride CM, Halabi S, Bepler G, et al. Maximizing the motivational impact of feedback of lung cancer susceptibility on smokers’ desire to quit. *J Health Commun*. 2000;5(3):229-241. doi:10.1080/10810730050131406

86. McBride CM, Bepler G, Lipkus IM, et al. Incorporating genetic susceptibility feedback into a smoking cessation program for African-American smokers with low income. *Cancer Epidemiol Biomark Prev Publ Am Assoc Cancer Res Cosponsored Am Soc Prev Oncol*. 2002;11(6):521-528.

87. Meagher KM, Curtis SH, Borucki S, et al. Communicating unexpected pharmacogenomic results to biobank contributors: A focus group study. *Patient Educ Couns*. 2021;104(2):242-249. doi:10.1016/j.pec.2020.08.023

88. Monnard K, Benjamins MR, Hirschtick JL, Castro M, Roesch PT. Co-Creation of Knowledge: A Community-Based Approach to Multilevel Dissemination of Health Information. *Health Promot Pract*. 2021;22(2):215-223. doi:10.1177/1524839919865228

89. Muse ED, Chen SF, Liu S, et al. Response to Polygenic Risk: Results of the MyGeneRank Mobile Application-Based Coronary Artery Disease Study. Published online April 28, 2021. doi:10.1101/2021.04.26.21256141

90. Ndase P, Celum C, Campbell J, et al. Successful discontinuation of the placebo arm and provision of an effective HIV prevention product after a positive interim efficacy result: the partners PrEP study experience. *J Acquir Immune Defic Syndr 1999*. 2014;66(2):206-212. doi:10.1097/QAI.0000000000000141

91. Nestor JG, Marasa M, Milo-Rasouly H, et al. Pilot Study of Return of Genetic Results to Patients in Adult Nephrology. *Clin J Am Soc Nephrol CJASN*. 2020;15(5):651-664. doi:10.2215/CJN.12481019

92. Nickerson J, Lee E, Nedelman M, Aurora RN, Krieger A, Horowitz CR. Feasibility of Portable Sleep Monitors to Detect Obstructive Sleep Apnea (OSA) in a Vulnerable Urban Population. *J Am Board Fam Med*. 2015;28(2):257-264. doi:10.3122/jabfm.2015.02.140273

93. Norelli J, Zlotorzynska M, Sanchez T, Sullivan PS. Scaling Up CareKit: Lessons Learned from Expansion of a Centralized Home HIV and Sexually Transmitted Infection Testing Program. *Sex Transm Dis*. 2021;48(8S):S66-S70. doi:10.1097/OLQ.0000000000001473

94. Nusbaum R, Leventhal KG, Hooker GW, et al. Translational genomic research: protocol development and initial outcomes following SNP testing for colon cancer risk. *Transl Behav Med*. 2013;3(1):17-29. doi:10.1007/s13142-012-0149-0

95. Oerlemans S, Arts LP, Horevoorts NJ, Van De Poll-Franse LV. “Am I normal?” The Wishes of Patients With Lymphoma to Compare Their Patient-Reported Outcomes With Those of Their Peers. *J Med Internet Res*. 2017;19(8):e288. doi:10.2196/jmir.7079

96. Ohneda K, Hiratsuka M, Kawame H, et al. A Pilot Study for Return of Individual Pharmacogenomic Results to Population-Based Cohort Study Participants. *JMA J*. 2022;5(2):177-189. doi:10.31662/jmaj.2021-0156

97. Ohneda K, Hamanaka Y, Kawame H, et al. Returning individual genomic results to population-based cohort study participants with BRCA1/2 pathogenic variants. *BREAST CANCER*. 2023;30(1):110-120. doi:10.1007/s12282-022-01404-7

98. Olson JE, Rohrer Vitek CR, Bell EJ, et al. Participant-perceived understanding and perspectives on pharmacogenomics: the Mayo Clinic RIGHT protocol (Right Drug, Right Dose, Right Time). *Genet Med*. 2017;19(7):819-825. doi:10.1038/gim.2016.192

99. Ondenge K, McLellan-Lemal E, Awuonda E, Angira F, Mills L, Thomas T. Disseminating results: community response and input on Kisumu breastfeeding study. *Transl Behav Med*. 2015;5(2):207-215. doi:10.1007/s13142-014-0303-y

100. Ottman R, Freyer C, Mefford H, Poduri A, Lowenstein D, Epilepsy Return Results Workshop. Return of individual results in epilepsy genomic research: A view from the field. *EPILEPSIA*. 2018;59(9):1635-1642. doi:10.1111/epi.14530

101. Patrick-Miller L, Egleston BL, Daly M, et al. Implementation and outcomes of telephone disclosure of clinical BRCA1/2 test results. *Patient Educ Couns*. 2013;93(3):413-419. doi:10.1016/j.pec.2013.08.009

102. Peltekova I, Buhas D, Stern L, Kirby E, Yusuf A, Elsabbagh M. Enhancing the Impact of Genomics Research in Autism through Integration of Research Results into Routine Care Pathways-A Case Series. *J Pers Med*. 2021;11(8). doi:10.3390/jpm11080755

103. Perovich LJ, Ohayon JL, Cousins EM, et al. Reporting to parents on children’s exposures to asthma triggers in low-income and public housing, an interview-based case study of ethics, environmental literacy, individual action, and public health benefits. *Environ Health*. 2018;17(1):48. doi:10.1186/s12940-018-0395-9

104. Plon SE, Peterson LE, Friedman LC, Richards CS. Mammography behavior after receiving a negative BRCA1 mutation test result in the Ashkenazim: A Community-based study. *Genet Med*. 2000;2(6):307-311. doi:10.1097/00125817-200011000-00001

105. Polka E, Childs E, Friedman A, et al. MCR: Open-Source Software to Automate Compilation of Health Study Report-Back. *Int J Environ Res Public Health*. 2021;18(11). doi:10.3390/ijerph18116104

106. Pulford DJ, Harter P, Floquet A, et al. Communicating BRCA research results to patients enrolled in international clinical trials: lessons learnt from the AGO-OVAR 16 study. *BMC Med Ethics*. 2016;17(1):63.

107. Quandt SA, Doran AM, Rao P, Hoppin JA, Snively BM, Arcury TA. Reporting pesticide assessment results to farmworker families: development, implementation, and evaluation of a risk communication strategy. *Environ Health Perspect*. 2004;112(5):636-642. doi:10.1289/ehp.6754

108. Ramirez-Vazquez R, Gonzalez-Rubio J, Arribas E, Najera A. Characterisation of personal exposure to environmental radiofrequency electromagnetic fields in Albacete (Spain) and assessment of risk perception. *Environ Res*. 2019;172(ei2, 0147621):109-116. doi:10.1016/j.envres.2019.02.015

109. Richards CS, Ward PA, Roa BB, et al. Screening for 1 85delAG in the Ashkenazim. *Am J Hum Genet*. Published online 1997.

110. Rohlman D, Donatuto J, Heidt M, et al. A Case Study Describing a Community-Engaged Approach for Evaluating Polycyclic Aromatic Hydrocarbon Exposure in a Native American Community. *Int J Environ Res Public Health*. 2019;16(3). doi:10.3390/ijerph16030327

111. Rowley SM, Mascarenhas L, Devereux L, et al. Population-based genetic testing of asymptomatic women for breast and ovarian cancer susceptibility. *Genet Med*. 2019;21(4):913-922. doi:10.1038/s41436-018-0277-0

112. Salvi N, Itta K, Lachyan A, et al. Experiences of sharing results of community based serosurvey with participants in a district of Maharashtra, India. *PLOS ONE*. 2022;17(8). doi:10.1371/journal.pone.0271920

113. Samaan Z, Schulze KM, Middleton C, et al. South Asian Heart Risk Assessment (SAHARA): Randomized Controlled Trial Design and Pilot Study. *JMIR Res Protoc*. 2013;2(2):e33. doi:10.2196/resprot.2621

114. Sanderson SC, O’Neill SC, White DB, et al. Responses to Online *GSTM1* Genetic Test Results among Smokers Related to Patients with Lung Cancer: A Pilot Study. *Cancer Epidemiol Biomarkers Prev*. 2009;18(7):1953-1961. doi:10.1158/1055-9965.EPI-08-0620

115. Sandhaus S, Kaufmann D, Ramirez-Andreotta M. Public Participation, Trust and Data Sharing: Gardens as Hubs for Citizen Science and Environmental Health Literacy Efforts. *Int J Sci Educ Part B Commun Public Engagem*. 2019;9(1):54-71. doi:10.1080/21548455.2018.1542752

116. Savatt J, Azzariti D, Faucett W, et al. ClinGen’s GenomeConnect registry enables patient-centered data sharing. *Hum Mutat*. 2018;39(11):1668-1676. doi:10.1002/humu.23633

117. Schollaert C, Alvarez M, Gillooly S, et al. Reporting Results of a Community-Based In-Home Exposure Monitoring Study: Developing Methods and Materials. *Prog Community Health Partnersh Res Educ Action*. 2021;15(1):117-125. doi:10.1353/cpr.2021.0011

118. Schwartz MD, Valdimarsdottir HB, Peshkin BN, et al. Randomized Noninferiority Trial of Telephone Versus In-Person Genetic Counseling for Hereditary Breast and Ovarian Cancer. *J Clin Oncol*. 2014;32(7):618-626. doi:10.1200/JCO.2013.51.3226

119. Shaibi G, Kullo I, Singh D, et al. Returning genomic results in a Federally Qualified Health Center: the intersection of precision medicine and social determinants of health. *Genet Med*. 2020;22(9):1552-1559. doi:10.1038/s41436-020-0806-5

120. Sharff ME, DeMarco TA, Mays D, et al. Parenting Through Genetic Uncertainty: Themes in the Disclosure of Breast Cancer Risk Information to Children. *Genet Test Mol Biomark*. 2012;16(5):376-382. doi:10.1089/gtmb.2011.0154

121. Shepherd R, Forrest L, Tutty E, et al. Unselected Women’s Experiences of Receiving Genetic Research Results for Hereditary Breast and Ovarian Cancer: A Qualitative Study. *Genet Test Mol Biomark*. 2021;25(12):741-748. doi:10.1089/gtmb.2021.0115

122. Siegfried JD, Morales A, Kushner JD, et al. Return of Genetic Results in the Familial Dilated Cardiomyopathy Research Project. *J Genet Couns*. 2013;22(2):164-174. doi:10.1007/s10897-012-9532-8

123. Smith KR, West JA, Croyle RT, Botkin JR. Familial context of genetic testing for cancer susceptibility: moderating effect of siblings’ test results on psychological distress one to two weeks after BRCA1 mutation testing. *Cancer Epidemiol Biomark Prev Publ Am Assoc Cancer Res Cosponsored Am Soc Prev Oncol*. 1999;8(4 Pt 2):385-392.

124. Sommer GJ, Wang TR, Epperson JG, et al. At‐home sperm testing for epidemiologic studies: Evaluation of the Trak male fertility testing system in an internet‐based preconception cohort. *Paediatr Perinat Epidemiol*. 2020;34(5):504-512. doi:10.1111/ppe.12612

125. South A, Joharatnam-Hogan N, Purvis C, et al. Testing approaches to sharing trial results with participants: The Show RESPECT cluster randomised, factorial, mixed methods trial. *PLOS Med*. 2021;18(10):e1003798. doi:10.1371/journal.pmed.1003798

126. Spagnolo J, Gautier L, Champagne F, et al. Reflecting on knowledge translation strategies from global health research projects in Tunisia and the Republic of Côte d’Ivoire. *Int J Public Health*. 2020;65(9):1559-1570. doi:10.1007/s00038-020-01502-3

127. Stefansdottir V, Thorolfsdottir E, Hognason H, et al. Web-based return ofBRCA2research results: one-year genetic counselling experience in Iceland. *Eur J Hum Genet*. 2020;28(12):1656-1661. doi:10.1038/s41431-020-0665-1

128. Streeten EA, See VY, Jeng LBJ, et al. KCNQ1 and Long QT Syndrome in 1/45 Amish: The Road From Identification to Implementation of Culturally Appropriate Precision Medicine. Regeneron Genetics Center*, ed. *Circ Genomic Precis Med*. 2020;13(6):e003133. doi:10.1161/CIRCGEN.120.003133

129. Tabor HK, Jamal SM, Yu JH, et al. My46: a Web-based tool for self-guided management of genomic test results in research and clinical settings. *Genet Med*. 2017;19(4):467-475. doi:10.1038/gim.2016.133

130. Tomsho KS, Basra K, Rubin SM, et al. Community reporting of ambient air polychlorinated biphenyl concentrations near a Superfund site. *Environ Sci Pollut Res*. 2018;25(17):16389-16400. doi:10.1007/s11356-017-0286-6

131. Tzovaras B, Angrist M, Arvai K, et al. Open Humans: A platform for participant-centered research and personal data exploration. *GIGASCIENCE*. 2019;8(6). doi:10.1093/gigascience/giz076

132. van de Poll-Franse L, Horevoorts N, Schoormans D, et al. Measuring Clinical, Biological, and Behavioral Variables to Elucidate Trajectories of Patient-Reported Outcomes: The PROFILES Registry. *JNCI-J Natl CANCER Inst*. 2022;114(6):800-807. doi:10.1093/jnci/djac047

133. Van Dijk S, Otten W, Timmermans DRM, et al. What’s the message? Interpretation of an uninformative BRCA1/2 test result for women at risk of familial breast cancer. *Genet Med*. 2005;7(4):239-245. doi:10.1097/01.GIM.0000159902.34833.26

134. Van Oostrom I, Meijers-Heijboer H, Duivenvoorden HJ, et al. Comparison of individuals opting for BRCA1/2 or HNPCC genetic susceptibility testing with regard to coping, illness perceptions, illness experiences, family system characteristics and hereditary cancer distress. *Patient Educ Couns*. 2007;65(1):58-68. doi:10.1016/j.pec.2006.05.006

135. Vassy JL, Lautenbach DM, McLaughlin HM, et al. The MedSeq Project: a randomized trial of integrating whole genome sequencing into clinical medicine. *Trials*. 2014;15(1):85. doi:10.1186/1745-6215-15-85

136. Von Behren J, Wong M, Morales D, Reynolds P, English P, Solomon G. Returning Individual Tap Water Testing Results to Research Study Participants after a Wildfire Disaster. *Int J Environ Res Public Health*. 2022;19(2). doi:10.3390/ijerph19020907

137. Wakefield CE, Thorne H, Kirk J, Niedermayr E, Doolan EL, Tucker K. Improving mutation notification when new genetic information is identified in research: a trial of two strategies in familial breast cancer. *Genet Med*. 2013;15(3):187-194. doi:10.1038/gim.2012.115

138. Widén E, Junna N, Ruotsalainen S, et al. How Communicating Polygenic and Clinical Risk for Atherosclerotic Cardiovascular Disease Impacts Health Behavior: an Observational Follow-up Study. *Circ Genomic Precis Med*. 2022;15(2). doi:10.1161/CIRCGEN.121.003459

139. Wiggins S, Whyte P, Huggins M, et al. The psychological consequences of predictive testing for Huntington’s disease. Canadian Collaborative Study of Predictive Testing. *N Engl J Med*. 1992;327(20):1401-1405. doi:10.1056/NEJM199211123272001

140. Williams JL, Rahm AK, Zallen DT, et al. Impact of a Patient‐Facing Enhanced Genomic Results Report to Improve Understanding, Engagement, and Communication. *J Genet Couns*. 2018;27(2):358-369. doi:10.1007/s10897-017-0176-6
