# Supplementary material for: Developing Requirements for a Standardized System to Return Individual Research Results Back to Study Participants: Narrative Review
Source: Interact J Med Res. 2025 Aug 18;14:e65606. doi: 10.2196/65606 (PMC12387377; doi:10.2196/65606)
Supplement: Multimedia Appendix 4 [file ijmr-v14-e65606-s004.docx]

| **Study** | **Authority** | **Accuracy** | **Coverage** | **Objectivity** | **Date** | **Significance** |
| --- | --- | --- | --- | --- | --- | --- |
| **Boronow, 2018; Perovich, 2017** | **✓** | **✓** | **✓** | **✓** | **✓** | **✓** |
| **Cope, 2023** | **✓** | **✓** | **✓** | **✓** | **✓** | **✓** |
| **Gilbert, 2022** | **✓** | **✓** | **✓** | **?** | **✓** | **✓** |
| **Ohneda, 2022** | **✓** | **✓** | **✓** | **✓** | **✓** | **✓** |
| **Polka, 2021** | **✓** | **?** | **✓** | **✓** | **✓** | **✓** |
| **Savatt, 2018** | **✓** | **✓** | **✓** | **✓** | **✓** | **?** |
| **van de Poll-Franse, 2022** | **✓** | **✓** | **✓** | **✓** | **✓** | **✓** |
